# Preparing Australasian medical students for environmentally sustainable health care

**DOI:** 10.5694/mja2.51439

**Published:** 2022-03-01

**Authors:** Diana L Madden, Graeme L Horton, Michelle McLean

**Affiliations:** ^1^ University of Notre Dame, Australia Sydney NSW; ^2^ University of Newcastle Newcastle NSW; ^3^ Bond University Gold Coast QLD

**Keywords:** Climate change, Global warming, Curriculum, Education, medical

Medical educators and representatives of medical student associations in Australia and New Zealand are collaborating on an initiative on climate change and health in medical education

Climate change and environmental degradation are harming the health of Australians and New Zealanders and pose a serious challenge to our health care systems.^1^, ^2^ The World Health Organization in 2019 identified climate change and air pollution as the top threat to human health globally;^3^ a threat clearly visible in the sustained air pollution over south‐eastern Australia from bushfire smoke during the 2019–20 bushfire season. The health consequences of the bushfires and the associated prolonged, hazardous air quality prompted the Australian Medical Association and three medical colleges to declare climate change a public health emergency.^4^ Health care systems also contribute to climate change and environmental degradation. In Australia, health care contributes 7% of the country’s total carbon emissions and produces considerable waste which is either incinerated or sent to landfill.^5^ Despite these environmental challenges, there has been little response by medical programs to prepare medical graduates to manage the health impacts of climate change, and to practise environmentally sustainable health care. In 2018, we described an initiative to support medical educators and medical student organisations to work collaboratively to develop proposed learning objectives, curricula and learning resources addressing the health effects of climate change.^6^ This article describes the development of the model graduate outcome statements and learning objectives which have been shared with all medical schools in Australasia.

## Background to development

Australia and New Zealand have accreditation standards for the education of the regulated health professions. For the medical profession in Australia and New Zealand, standards are maintained by the Australian Medical Council (AMC), including for the 23 university medical schools offering medical programs.^7^ The standards for medical programs include graduate outcome statements, which describe what medical students should be able to do at graduation. Forty statements are grouped into four domains of practice (science and scholarship, clinical practice, health and society, professionalism and leadership) and these inform the development of medical curricula. Accreditation standards are used to ensure quality and drive change in practice. The current accreditation standards and graduate outcome statements do not mention climate change or environmentally sustainable health care. Consequently, developing suitable amendments to these standards and statements, with which medical educators are already familiar, can help frame curriculum change.

A Working Group on Climate Change and Health was formed when medical educators from several Australian medical schools submitted a proposal to the Medical Deans Australia and New Zealand Medical Education Collaborative Committee that it explore the development of model learning objectives and learning resources regarding the impact of climate change on health.

To examine current environmentally sustainable health care education, the Working Group searched the literature, reviewed teaching experience, and benchmarked against relevant courses developed for medical students, including those developed by students for their peers.^8^, ^9^, ^10^, ^11^, ^12^, ^13^ Through this process, the Working Group identified five broad areas of learning to guide the development of curriculum resources including proposed graduate outcome statements and learning objectives:

▪ the evidence for anthropogenic climate change and how this relates to the environmental determinants of health;
▪ the impact of climate change on health;
▪ the impact of climate change on the health system;
▪ the environmental impact of the health sector and the elements of environmentally sustainable health care; and
▪ creating change, both intra‐ and inter‐sectorally, through practice, advocacy and leadership.

The current 2012 AMC graduate outcome statements were mapped against these five areas to determine whether they were sufficient or whether amendments or new statements were required. A minimal change approach was adopted, mirroring changes to the accreditation standards adopted in 2018 by the United Kingdom General Medical Council.^14^ Rather than referring specifically to climate change, the General Medical Council focuses on the environment as a determinant of health. By 2020, all UK medical schools were required to ensure that graduating doctors were able to apply the knowledge and principles of environmentally sustainable health care to their practice (graduate outcome 25).

The Working Group considered that, by focusing on the environment as a determinant of health, climate change would be addressed. As sustainable health care in Australia usually refers to financial sustainability, to avoid confusion, the term environmental sustainability is used. The amendments proposed by the Working Group also introduce the concept that decisions taken today in the care of patients and populations should not compromise the health of future generations.

## Graduate outcome statements: proposed amendments

The proposed amendments to the graduate outcome statements represent modest changes to the existing statements. Fourteen statements were identified: ten were modified to be aligned and four did not require amendment. Statements have been identified in all four AMC domains of practice. The Box presents the statements with the suggested amendments.

Box 1 **Australian Medical Council graduate outcome statements for primary medical programs for all four domains of practice,^7^ with proposed amendments in italics, aligned with preparing medical students to practise environmentally sustainable health care (learning objectives to support each statement are included) — prepared by the Climate Change and Health Working Group of Medical Deans Australia and New Zealand**

**Table:** 

Graduate outcome statement	Learning objective aligned with graduate outcome statement
**Domain 1: Science and scholarship — the medical graduate as scientist and scholar**	
On entry to professional practice, Australian and New Zealand graduates are able to:	
1.1 Demonstrate an understanding of established and evolving biological, *environmental*, clinical, epidemiological, social and behavioural sciences.	▪ Outline the dependence of human health on global and local ecological systems which supply clean air, clean water and nutritious food and the Earth systems that provide a stable climate.^10^ ▪ Discuss the contribution of human activity to global and local environmental changes such as climate change, and biodiversity loss and resource depletion in land and marine environments.^10^ ▪ Describe the mechanisms by which human health is affected by environmental change; eg, exposure to extreme weather, change in disease vectors, migration and decreasing food and water security.^10^
**Domain 2: Clinical practice — the medical graduate as practitioner**	
On entry to professional practice, Australian and New Zealand graduates are able to:	
2.2 Elicit an accurate, organised and problem‐focused medical history, including family, social, occupational, *environmental* and lifestyle features, from the patient, and other sources.	▪ Take a focused occupational and environmental history.^10^
2.5 Select and justify common investigations, with regard to the pathological basis of disease, utility, safety, cost‐effectiveness *and environmental sustainability*, and interpret their results.	▪ Propose ways to practise medicine sustainably by considering the environmental impact of investigations used in the diagnosis and management of patients.*
2.10 Integrate prevention, early detection, health maintenance and chronic condition management where relevant to clinical practice.	▪ Prevent, diagnose and treat the adverse health effects attributed to climate change and environmental causes.^15^ ▪ Propose ways to practise medicine sustainably by considering what models of care could reduce the environmental impact of best practice care and service delivery to patients.*
2.11 Prescribe medications safely, effectively, economically *and sustainably* using objective evidence. Safely administer other therapeutic agents including fluid, electrolytes, blood products and selected inhalational agents.	▪ Propose ways to practise medicine sustainably by considering the environmental impact of medications and other treatments in prescribing decisions.*
**Domain 3: Health and society — the medical graduate as a health advocate**	
On entry to professional practice, Australian and New Zealand graduates are able to:	
3.1 Accept responsibility to protect and advance the health and wellbeing of individuals, communities and populations *now and for future generations*.	▪ Identify the role of health care professionals in advocating for policies and infrastructure that promote the availability, accessibility and uptake of healthy and environmentally sustainable behaviours^13^ (this objective also aligns with 3.3). ▪ Discuss ethical tensions between allocating resources to individual patients and protecting the environment upon which the health of the wider community depends^10^ (this objective also aligns with 4.4).
3.2 Explain factors that contribute to the health, illness, disease and success of treatment of populations, including issues relating to health inequities and inequalities, diversity of cultural, spiritual and community values, and *the* socio‐economic and *environmental determinants of health*.	▪ Describe features of a health‐promoting local environment, in community and health care settings, to include access to green spaces, clean air, and an active travel infrastructure^10^ (this objective also aligns with 3.9). ▪ Explain the concept of “health co‐benefits” by considering how lifestyle choices can promote both patient wellbeing and a healthy environment.^13^ ▪ Explain how the health impacts of environmental change are distributed unequally within and between populations, and the disparity between those most responsible and those most affected by change^10^ (this objective also aligns with 3.9).
3.3 Communicate effectively in wider roles including health advocacy, teaching, assessing and appraising.	▪ Inform patients and the public about the need to take action on climate change and the health benefits of an environmentally sustainable society.^13^ ▪ Evaluate their work environment for the level of environmental sustainability and promote environmentally sustainable health care practices.^10^ ▪ Identify the role of health care professionals in advocating for policies and infrastructure that promote the availability, accessibility and uptake of healthy and environmentally sustainable behaviours^13^ (this objective also aligns with 3.1).
3.6 Describe a systems approach to *understanding health and* improving the quality and safety of health care.	▪ Identify the vulnerabilities of health services and health facilities to climate change and extreme weather events and how these risks can be minimised and prepared for.^15^ ▪ Compare the carbon footprint of the Australian and New Zealand health care systems with those of other countries and describe the major contributors to each* (this objective also aligns with 3.7). ▪ Participate in quality improvement processes that aim to measure and improve the environmental sustainability of a health care service, considering not only the intended health benefits but also the associated financial, social and environmental costs^13^ (this objective also aligns with 3.7 and 4.2). ▪ Identify ways to improve the environmental sustainability of health care systems by reducing the carbon footprint through individual practice, health service management and the design of care systems^10^ (this objective also aligns with 3.7 and 4.3).
3.7 Understand and describe the roles and relationships between health agencies and services, and explain the principles of efficient, *environmentally sustainable* and equitable allocation of finite resources, to meet individual, community and national health needs.	▪ Explain how trends in climate change may affect capacity to provide health care in the future^10^ (this objective also aligns with 3.9). ▪ Understand the role of international, national and state policy frameworks in addressing health risks of climate change.^13^ ▪ Explain the contribution of the Sustainable Development Goals to addressing the socio‐economic and environmental determinants of health in Australasia and the Pacific* (this objective also aligns with 3.9). ▪ Compare the carbon footprint of the Australian and New Zealand health care systems with those of other countries and describe the major contributors to each* (this objective also aligns with 3.6). ▪ Participate in quality improvement processes that aim to measure and improve the environmental sustainability of a health care service, considering not only the intended health benefits but also the associated financial, social and environmental costs^13^ (this objective also aligns with 3.6 and 4.2). ▪ Identify ways to improve the environmental sustainability of health care systems by reducing the carbon footprint through individual practice, health service management and the design of care systems^10^ (this objective also aligns with 3.6 and 4.3).
3.9 Demonstrate an understanding of global health issues and determinants of health and disease including their relevance to health care delivery in Australia and New Zealand and the broader Western Pacific region. *Understand the contribution of the Sustainable Development Goals to addressing the socio‐economic and environmental determinants of health*.	▪ Describe features of a health‐promoting local environment, in community and health care settings, to include access to green spaces, clean air, and an active travel infrastructure^10^ (this objective also aligns with 3.2). ▪ Explain how the health impacts of environmental change are distributed unequally within and between populations, and the disparity between those most responsible and those most affected by change^10^ (this objective also aligns with 3.2). ▪ Explain how trends in climate change may affect capacity to provide health care in the future^10^ (this objective also aligns with 3.7). ▪ Explain the contribution of the Sustainable Development Goals to addressing the socio‐economic and environmental determinants of health in Australasia and the Pacific* (this objective also aligns with 3.7).
**Domain 4: Professionalism and leadership — the medical graduate as a professional and leader**	
On entry to professional practice, Australian and New Zealand graduates are able to:	
4.2 Demonstrate professional values including commitment to high quality clinical standards, compassion, empathy and respect for all patients. Demonstrate the qualities of integrity, honesty, leadership and partnership to patients, the profession and society.	▪ Participate in quality improvement processes that aim to measure and improve the environmental sustainability of a health care service, considering not only the intended health benefits but also the associated financial, social and environmental costs^13^ (this objective also aligns with 3.6 and 3.7).
4.3 Describe the principles and practice of professionalism and leadership in health care *that support the health and wellbeing of individuals, communities and populations now and for future generations*.	▪ Identify ways to improve the environmental sustainability of health care systems by reducing the carbon footprint through individual practice, health service management and the design of care systems^10^ (this objective also aligns with 3.6 and 3.7).
4.4 Explain the main principles of ethical practice and apply these to learning scenarios in clinical practice. Communicate effectively about ethical issues with patients, family and other health care professionals.	▪ Discuss ethical tensions between allocating resources to individual patients and protecting the environment upon which the health of the wider community depends^10^ (this objective also aligns with 3.1).

* Source: Climate Change and Health Working Group of Medical Deans Australia and New Zealand.

Learning objectives regarding climate change and health in medical and health professional education that support each statement have been identified from recently published literature.^8^, ^9^, ^10^, ^11^, ^12^, ^13^ Many of these learning objectives have been through a process of validation. In some instances, a learning objective can be aligned with more than one statement. In 2018, when the Working Group was examining this literature, the literature was restricted, reflecting the lack of activity in health professions education to prepare the health workforce for climate change. The sources were therefore supplemented by the Core Climate and Health Competencies for Health Professionals developed by a global consortium convened through Columbia University.^15^ In some instances, it was necessary for the Working Group to develop new learning objectives. The Box also presents these learning objectives and indicates the statements they support.

## Distribution and next steps

The proposed graduate outcome statements and learning objectives were circulated to the Deans of all medical schools in August 2018 for further consideration and as a potential curriculum resource when considering curriculum review. Resourcing changes to curricula is a major hurdle to the successful implementation of change.

However, the suggested amendments will only be sustained and embedded into practice across Australia and New Zealand if they are adopted by the AMC and become part of the Standards for Assessment and Accreditation of Primary Medical Programs. A review by the AMC of the accreditation standards for primary medical programs opened in 2021 and the consultation process for this review continues in 2022 (https://www.amc.org.au/review‐of‐accreditation‐standards‐for‐primary‐medical‐programs‐medical‐schools/). A submission has been provided to the consultation by the authors.

## Measuring and monitoring

From 2023, the annual *MJA–Lancet* Countdown report aims to report an indicator describing progress on the inclusion of environmental sustainability and the health impacts of climate change in medical curricula in Australia.^2^ The authors are looking to establish a research agenda to determine how this indicator could be introduced. Its purpose, who will use it, and what data will be collected and how, will be established with stakeholder involvement.

Medical educators internationally and other health professions in Australia have shown strong interest in this collaborative approach to aligning the education of health professional graduates with environmentally sustainable health care.

## Conclusion

Through this initiative, the collaboration of medical educators and medical student associations in Australasia has shared with all medical schools an approach to incorporating the health effects of climate change and the environmental impact of health care within medical curricula. The work is stimulating a dialogue within faculties and among medical students. The process recognises the essential contribution of the AMC Standards to secure and drive these changes to prepare medical graduates to practise environmentally sustainable health care. Amending the AMC graduate outcome statements to frame curricula change, and working through the network of medical schools, enables system level change in medical curricula. New patterns of disease are already emerging and medical practice is evolving as health care systems are challenged to become more environmentally sustainable; doctors and other health professionals are increasingly using their influence to help society transition to an environmentally sustainable future. Health educators have an essential part to play in preparing future doctors for these roles.

## Disclaimer

The statements or opinions expressed in this article reflect the views of the authors and do not represent the opinion or policies of Medical Deans Australia and New Zealand Inc.

## Competing interests

No relevant disclosures.

## Provenance

Commissioned; externally peer reviewed.
